# 开放性实验：二氧化硫的微萃取及智能手机比色法辅助的快速定量分析

**DOI:** 10.3724/SP.J.1123.2025.09027

**Published:** 2026-05-08

**Authors:** Liang ZHANG, Chang LIU, Jia ZHU, Jinghui ZHANG, Wei SHEN, Sheng TANG

**Affiliations:** 1.江苏科技大学环境与化学工程学院，江苏 镇江 212100; 1. School of Environmental and Chemical Engineering，Jiangsu University of Science and Technology，Zhenjiang 212100，China; 2.江苏科技大学粮食学院，江苏 镇江 212100; 2. School of Grain Science and Technology，Jiangsu University of Science and Technology，Zhenjiang 212100，China

**Keywords:** 智能手机比色法, 单滴微萃取, 二氧化硫, 光信号, 开放性实验, smartphone-based colorimetry, single-drop microextraction, sulfur dioxide, optical signal, open-ended experiment

## Abstract

开放性实验课程是培养本科生实践能力、创新意识与科学素养的重要环节。本项目设计并实施了以“智能手机比色法定量检测二氧化硫（SO_2_）气体污染物”为主题的开放性实验。在实验过程中，采用顶空单滴微萃取法富集样品中挥发的SO_2_，SO_2_与银纳米粒子在单滴体系中发生刻蚀反应并引发溶液颜色的信号变化，通过智能手机的比色测定，从而实现对SO_2_的定量分析。该实验引导学生运用智能手机作为新型的便携检测平台，启发其对各类科研工具的创新运用。实验设计不但涉及构建标准曲线、样品定量分析、比色法等基础分析化学实验基础知识与技能，还引入了样品微萃取、纳米材料显色反应等较为前沿的分析手段。该开放性实验课程可帮助本科生理解环境监测中比色分析技术应用的基本思路，使学生在实际科研情境中理解理论与技术的紧密联系。在实验过程中，学生不仅可以体验到科学探索的严谨性和创新性，还能感受到科学实验对环境保护、社会发展及国家科技进步的重要意义，从而在潜移默化中形成科学精神、社会责任感与专业使命感，实现专业素养与思政教育的深度融合。

## Abstract (en)

Open-ended experiment courses play a crucial role in cultivating practical skills， innovative thinking and scientific literacy of undergraduate students. In this project， an open-ended experiment was designed and implemented with the theme of quantitative detection of sulfur dioxide （SO_2_） gas pollutants using smartphone-based colorimetry. During the experiment， volatile SO₂ in samples was enriched using headspace single-drop microextraction. In the single-drop system， SO_2_ reacted with silver nanoparticles， inducing a distinct color change. SO_2_ concentration was quantified through colorimetric analysis using a smartphone as a portable detection platform. This experiment guided students to employ smartphones as a novel portable detection platform， inspiring the innovative application of diverse scientific tools. The design covered fundamental analytical chemistry skills， including calibration curve construction， sample quantification and colorimetric analysis. Advanced techniques， such as microextraction and nanoparticle-mediated color reactions， were also introduced. This open-ended experiment course enables undergraduates to understand the core principles of colorimetric analysis in environmental monitoring， fostering a comprehension of the close link between theory and practical techniques in real research contexts. Throughout the experiment， students experience both the rigor and creativity of scientific exploration， while recognizing the broader significance of scientific experimentation in environmental protection， societal development and national technological advancement. Consequently， the course facilitates the internalization of scientific spirit， social responsibility and professional mission， achieving a deep integration of professional competence with values-based education.

在当前高等教育仪器分析实验教学中，将实际检测问题（如环境监测）融入本科生开放实验课程，已成为培养学生实践能力、创新思维和跨学科综合素养的重要途径之一^［1，2］^。在众多的环境污染中，无机气体污染物作为一种重要的大气环境污染物，对区域空气质量和人体健康具有显著影响。实现对该类污染物的高效富集与准确检测对于环境空气质量的保障具有重要的社会意义^［3-5］^。在该类污染物检测中，样品前处理过程是影响检测结果准确性与可靠性的关键环节，合理的气体富集与提取步骤可有效降低基体干扰并提高检测灵敏度。因此，将环境样品前处理理念与方法引入本科实验教学，不仅对帮助学生理解“从样品到数据”的完整分析链条具有重要意义，而且能够激发学生的学习兴趣和科研热情，引导学生认识科学研究与社会发展、环境保护之间的密切关系，培养科学精神、社会责任感和服务国家发展的使命意识。

本实验以环境科学和化学学科本科生为主要教学对象，通过将智能手机比色法与顶空单滴微萃取（headspace single-drop microextraction，HS-SDME）法相结合^［6，7］^，实现对无机气体污染物的定量分析。在实验过程中，选择二氧化硫（SO_2_）作为目标物，SO_2_挥发后被含银纳米颗粒（Ag NPs）的微滴萃取富集，并在单滴体系内对Ag NPs产生刻蚀作用，液滴颜色随着Ag NPs微观结构的改变而发生变化，随后通过智能手机拍照取色获取光学信号并进行比色分析，最终实现样品中SO_2_的定量检测。该实验模式突破了对传统光学仪器的依赖，展示了纳米材料、分析化学技术与信息技术在环境检测中的融合与协同应用^［8］^，通过这一实验，学生不仅能直观理解前处理过程在提高检测准确性中的作用，拓展他们对智能手机作为便携式检测仪器的应用思路，同时还能掌握纳米材料在气体富集与反应识别中的分析原理。

本实验在教学设计上的创新性体现在3个方面：首先，以开放性实验形式鼓励学生自主设计实验方案和数据处理流程，强化学生的创新意识与独立思考能力；其次，将环境化学、纳米科学与智能便携分析平台有机融合，培养学生的跨学科综合应用能力与科研素养；最后，该实验操作简便、成本可控且具有较强的实时性，有助于学生在掌握现代环境检测技术的同时，深入理解其在环境样品分析、污染检测及社会可持续发展中的重要意义。通过该实验，学生能够系统掌握智能手机比色法与样品前处理技术的基本原理和应用方法，并在实践过程中培养问题分析能力、实验设计能力和团队协作能力，从而内化科学精神、社会责任感与专业使命意识。

## 1 实验原理

### 1.1 智能手机程序取色原理及颜色校正的数理统计

#### 1.1.1 物质显色的原理

物质的显色主要源于其对可见光的选择性响应。当可见光作用于物质时，物质会对光的吸收、反射、透射、折射和散射表现出特定规律。在可见光波段（波长约400~730 nm，对应波数约25 000~13 800 cm^-1^，能量约3.10~1.71 eV）内，若某一物质对该波段中的特定光谱成分发生选择性吸收，其所呈现的可见色即为被吸收光的互补色。

#### 1.1.2 高斯函数卷积运算（基于Retinex理论）

Retinex理论由Land于20世纪70年代提出，是一种基于颜色恒常性的图像处理理论^［9］^。该理论核心假设包括：真实世界本身无固有颜色，人类所感知的颜色源于光与物体的相互作用；所有颜色均可由红、绿、蓝三原色组成；三原色共同决定每个单位区域的颜色。基于此，Retinex理论能够对不同类型图像进行自适应增强，在动态范围压缩、细节增强及颜色校正等方面实现更好的平衡，相较于传统单一增强算法具备更强的适应性，因此得到广泛发展与应用。同时，高斯函数（滤波器）作为常用的图像特征提取方法，最初应用于模糊降噪和图像增强，近年来也被广泛用于光照分量提取和光源校正。在本研究中，我们基于Retinex理论，采用高斯滤波器与图像亮度值进行卷积运算，成功提取出图像光照分量，为后续GAMMA校正提供了基础。

#### 1.1.3 GAMMA校正

在物理世界中，光强度与亮度呈线性关系，即光强增加一倍，亮度也随之增加一倍。然而，早期显示器（如阴极射线管）输出亮度与电压并非线性，而是遵循非线性关系：亮度增加量与电压增加量的2.2次方成正比，2.2即为显示器的GAMMA值，现代显示器的GAMMA值也约为2.2。因此，人眼在真实环境下感知的亮度与显示器输出图像存在差异，GAMMA校正最早用于消除此差异。在检测分析的智能手机程序中，图像光源不均可能由相机自动曝光、环境因素或拍摄角度引起，严重干扰平行样品的分析。为避免图像失真，仅对HSV模型中的明度（*V*）分量进行处理，先通过高斯分布函数卷积提取*V*分量，再利用构建的GAMMA函数进行均衡化矫正，从而显著改善样品图像的平行性。

### 1.2 HS-SDME

HS-SDME是一种经典的液相微萃取技术，其基本特征是以最大限度减少有机溶剂的用量为前提，同时保持较高的样品与萃取相体积比，从而显著提升分析物的富集效率。该方法已广泛应用于液体与气体样品中挥发性和半挥发性组分的分离与富集。其基本原理在于：具有一定挥发性的目标组分可由样品基质向顶空气相迁移，并被悬挂于顶空中的微小萃取液滴捕获，实现对分析物的高效富集与选择性分离。

在本实验的教学设计中，学生将已知浓度梯度的亚硫酸钠溶液或实际样品置于带密封盖的离心管中，为获得目标气相SO_2_浓度，溶液配制时按化学计量关系预先确定硫的浓度（在酸化并完全转化为SO_2_的理想条件下，溶液中被质子化的硫氧阴离子与释放的SO_2_在物质的量上呈现1∶1的对应关系）。在每个离心管盖的内壁精确悬挂一滴含Ag NPs的萃取剂。该微小液滴在表面张力与分子间作用力的共同作用下可稳定悬挂于盖内（即使在将离心管倒置时亦不易脱落，仅在强烈振动或摇晃下可能脱落），向其中滴加盐酸溶液以提供足够的H^+^驱动反应定向进行，从而实现SO_2_的稳定释放。实验静置一定时间后，样品中释放的SO_2_逐步向顶空气相迁移，并被萃取相液滴高效吸收并富集。随后，学生按照规定步骤取下离心管盖，并将盖内萃取液滴作为分析对象，再采用本课题组自主开发的智能手机取色软件ICSO采集液滴的颜色参数，并据此完成对应光学信号的定量转换与数据记录^［10］^。ICSO的源代码，包括算法与模型，可从GitHub资源库获取，可访问地址为https://github.com/Jisencc/ICSO。

### 1.3 比色法的原理

比色法通过测定或比较有色物质的颜色深度来确定待测组分含量，是快速检测技术中最为常用的方法之一。基于智能手机的比色法流程如下：样品经化学反应或物理化学过程产生可见颜色变化，智能手机摄像头捕获颜色信息，并通过专用软件或应用程序提取颜色通道参数^［11-13］^。通过建立颜色参数与待测组分浓度之间的校准曲线，可实现快速、低成本、实时的定量分析。本实验中，SO_2_通过刻蚀Ag NPs引起纳米探针壳层折射率及核/壳尺寸比变化，促使纳米探针局域表面等离子体共振（localized surface plasmon resonance， LSPR）波长移动，并产生可被裸眼识别的颜色变化。随后，利用智能手机比色法程序软件提取检测区域的色度值，并结合线性或非线性回归方法建立色度信号与待测样品浓度的定量关系，从而实现SO_2_的定量检测（见图1）。

**图1:**
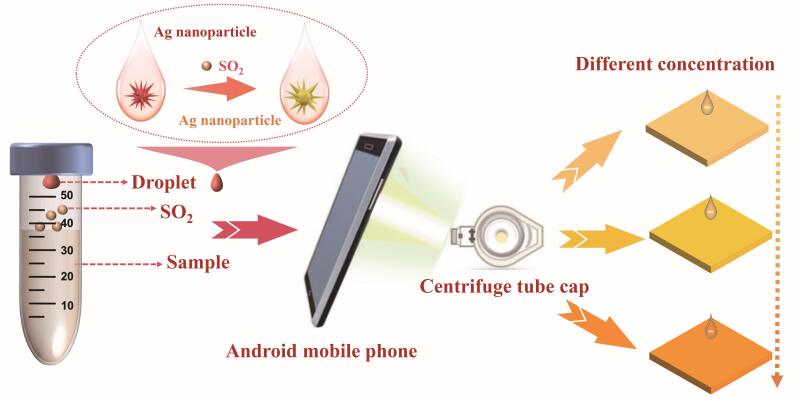
智能手机比色法与微萃取技术的SO_2_快速定量分析原理图

## 2 实验部分

### 2.1 仪器、试剂与材料

亚硫酸钠（Na_2_SO_3_，≥98%）、硼氢化钠（NaBH_4_，≥98%）、浓盐酸（37%，约为12 mol/L）、硝酸银（AgNO_3_，≥99.8%）均购于上海阿拉丁生化科技股份有限公司。

150 mL烧杯、100 mL烧杯和2.50 mL塑料离心管均购于成都市科隆化学品有限公司，锡箔纸购于南通帕达曼实验器材有限公司，磁力搅拌器购于上海科兴仪器有限公司，安卓智能手机（每个实验小组一部，由学生自行准备），并提前安装课题组自研ICSO取色软件。

### 2.2 Ag NPs的合成

配制100 mL浓度为0.10 mmol/L的AgNO_3_水溶液，将其置于150 mL烧杯中，在烧杯的杯壁外侧包上锡箔纸，置于磁力搅拌器上进行搅拌，随后将0.01 g NaBH_4_迅速加入到上述AgNO_3_水溶液中，并在烧杯顶部覆盖锡箔纸以遮光，持续避光搅拌10 min，反应结束后，获得呈浅黄色的Ag NPs溶液。反应过程可简化表示如下：

Ag^+^+BH_4_^-^+H_2_O→Ag^0^+H_3_BO_3_+H_2_ （1）

其中，Ag^0^以纳米颗粒形式分散于水中，形成稳定的浅黄色溶液

### 2.3 亚硫酸钠溶液的配制

亚硫酸钠系列标准溶液按以下方法配制：首先，称取126 mg亚硫酸钠于150 mL烧杯中，加入100 mL超纯水，制备得到10 mmol/L标准溶液。在此基础上通过稀释制备不同浓度梯度的溶液：将4 mL 10 mmol/L标准溶液稀释至40 mL配制1 mmol/L溶液；取3.20、2.00、1.60、0.80 mL 10 mmol/L标准溶液，分别稀释至40 mL配制得到对应浓度分别为0.80、0.50、0.40、0.20 mmol/L的溶液；将8 mL 0.50 mmol/L溶液稀释至40 mL配制0.10 mmol/L溶液。各溶液充分混合以确保均一性。

### 2.4 盐酸溶液的配制

0.50 mol/L盐酸溶液的配制：取市售浓盐酸4.2 mL于150 mL的烧杯中，加入95.8 mL超纯水，混合均匀。

### 2.5 单滴微萃取及比色测定

取9支2.50 mL塑料离心管，分别加入500 μL浓度为1、0.80、0.50、0.40、0.20与0.10 mmol/L的亚硫酸钠溶液，在每支塑料离心管盖中心处滴加10 μL Ag NPs溶液，然后向每支离心管中滴加1 mL 0.50 mol/L盐酸溶液并盖上离心管盖。由于表面张力的作用，单滴将附着在盖上，并实现顶空状态（图1）。其中，空白（只滴加10 μL Ag NPs）以及含有0.10、0.20、0.40、0.80、1 mmol/L亚硫酸钠的离心管作为标准品，含有0.50 mmol/L亚硫酸钠的离心管作为样品（样品设3份平行）。

在静置萃取80 min后，于通风橱桌面铺设一张A4纸，并将9支塑料离心管的盖子剪下，按浓度梯度排列于A4纸上，其中空白单滴与单滴萃取1、0.5、0.1 mmol/L亚硫酸钠溶液后形成的单滴（共6个样品）如图2所示。排列完成后，使用智能手机摄像头拍摄检测区域图像，并利用ICSO软件提取色度值^［14］^，通过建立标准样品浓度与色度值之间的线性关系，由待测样品的色度值推算其浓度，实现定量分析。

**图2:**
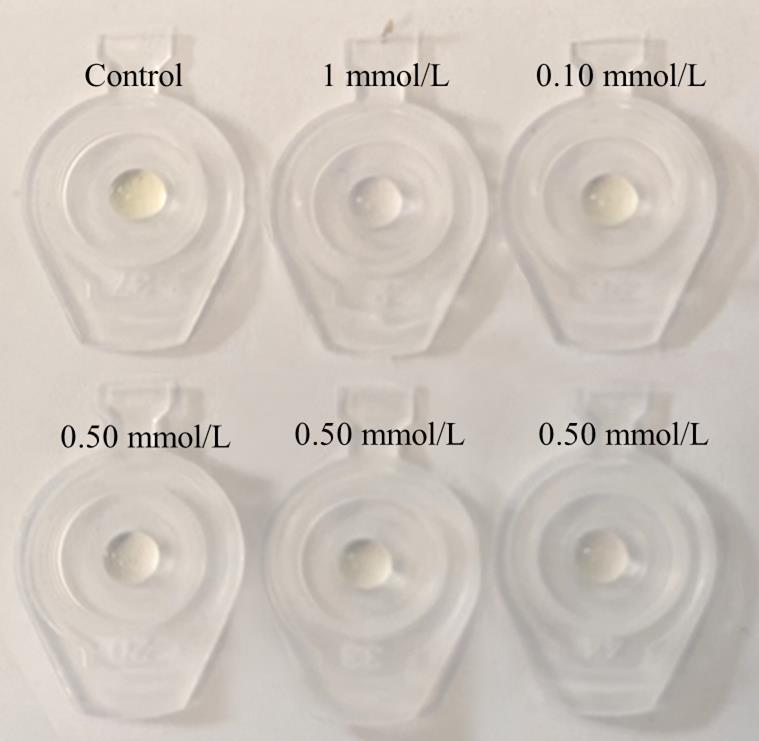
单滴萃取不同浓度亚硫酸钠溶液后形成的单滴排列图

## 3 结果与讨论

### 3.1 智能手机取色结果

图3a展示了单滴对不同浓度亚硫酸钠样品萃取后智能手机拍照得到的取色图。随着亚硫酸钠浓度的逐步升高，溶液中可释放的SO_2_含量相应增加，从而引发Ag NPs微小液滴的颜色变化，因此，单滴样品的表观颜色呈现出清晰的浓度依赖性差异。尽管0.10 mmol/L与1 mmol/L条件下的颜色差异并不显著，但0 mmol/L与0.10 mmol/L之间已表现出可辨识的颜色变化，这一现象清晰地证明了Ag NPs能够对由亚硫酸钠溶液释放的SO_2_产生响应，体现了该方法在低浓度区间内的高灵敏性。

Ag+SO_2_+O_2_+H_2_O→Ag_2_S+SO_4_^2-^+H^+^ (2)

**图3:**
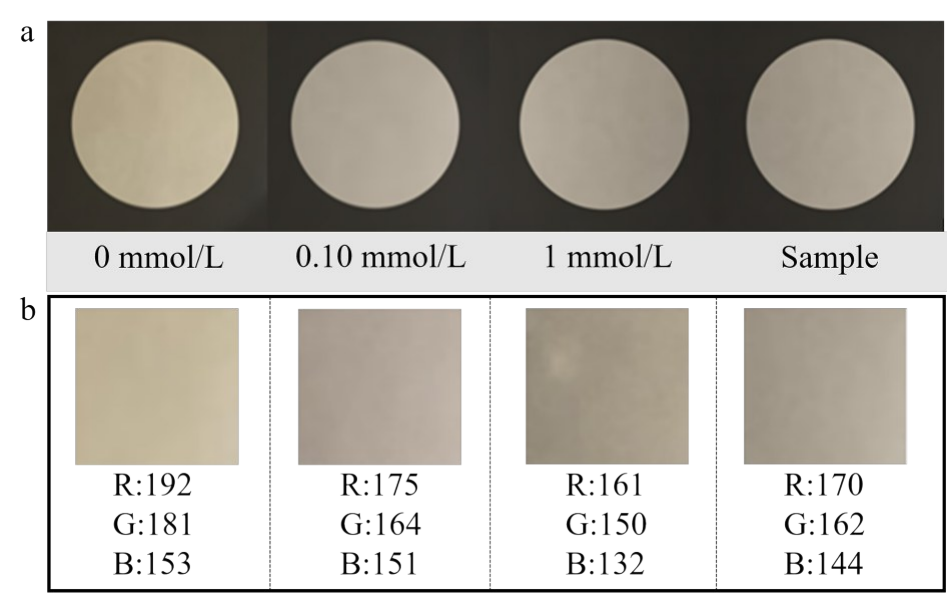
（a）智能手机取色图和（b）取色分析结果图

反应过程中，Ag NPs的表面逐渐被SO_2_刻蚀并生成Ag_2_S，导致其LSPR峰发生红移甚至消失，从而引发颜色变化。

基于智能手机比色分析方法对不同浓度样品的取色区域进行了定量分析^［7，10，15-18］^，其中4个样品的取色结果展示于图3b，获得颜色强度数据。随后，软件根据标准溶液的数据，自动生成了红、绿、蓝（RGB）三通道的标准曲线。最终，依据此标准曲线对样品进行取色分析，从而获得其浓度信息。

### 3.2 3种颜色的标准曲线

依据手机取色法获得的RGB数据绘制标准曲线（图4）可知，红色通道的信号强度对SO_2_浓度变化最为敏感，而蓝色通道的响应则相对平缓。这一显著差异源于体系中Ag NPs在刻蚀过程中的LSPR特性变化。初始溶液中的Ag NPs呈黄色，对可见光三原色均具有一定吸收，但其LSPR峰主要分布于红光波段。当SO_2_参与刻蚀反应后，纳米粒子的平均尺寸减小、形貌发生重构，同时其表面电子结构及化学环境亦随之改变，进而引起LSPR峰位的蓝移并伴随吸收强度的变化。鉴于黄色体系对红光区域的吸收变化最为敏感，粒径与表面状态的细微调控即可导致红光吸收产生显著响应，因此，在RGB色度分析中，红色通道表现出更优的线性响应特征。而绿光与蓝光波段的吸收变化相对有限，其对应通道对浓度变化的响应敏感度相对较低。

**图4:**
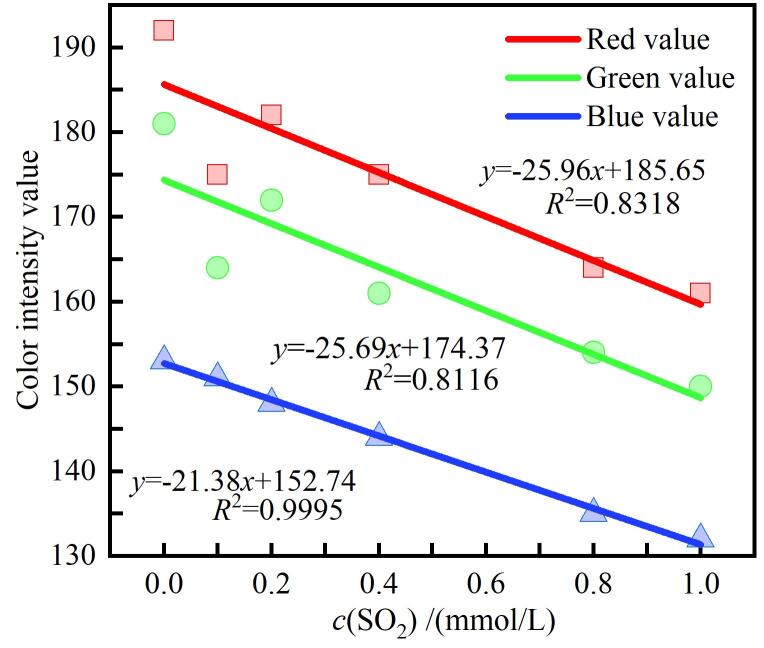
RGB 3种颜色的颜色强度随浓度变化的标准曲线

然而，对红光与绿光通道线性拟合结果的进一步分析表明，其相关性低于理论预期，说明除材料本征光学响应外，实验操作及检测条件亦对数据准确性产生了显著影响。检测过程中，若样品密封不充分，可能因微量蒸发或局部浓度波动引起体系光学特性的偏移，从而引入系统误差^［19］^。此外，基于智能手机的取色方法对实验条件具有较高的敏感性，包括光照环境、入射角度、测量距离及采集位置等因素，上述参数的轻微变化即可导致入射光强及其空间分布发生改变，尤其在低浓度区间，信噪比较低，系统性偏差易被进一步放大，进而使拟合关系偏离理想线性行为。除此之外，智能手机相机图像传感器的光谱响应特性、自动白平衡、曝光调节算法与JPEG（joint photographic experts group）压缩过程中引入的非线性失真，都可能对原始RGB数据造成潜在影响，使观测到的强度变化与实际吸收过程存在一定偏差。上述多重因素的共同作用最终导致红光和绿光通道的拟合结果表现出一定程度的离散性。

为提高线性拟合度与检测结果的可靠性，应从光学条件控制、实验操作规范与数据采集处理3个方面进行系统优化。首先，应确保检测光源与测量几何条件的可复现性，可通过引入光学暗箱或采用恒定照度的发光二极管光源以有效消除外界干扰^［20，21］^。同时，应固定相机与样品之间的距离与角度，并借助标准白板或色卡进行白平衡与光强校正，以最大限度减少系统误差。其次，样品的制备与保存条件应严格受控，尤其需确保离心管或反应容器的良好密封性，以避免因溶剂蒸发、气体逸散或温度波动引起的有效浓度漂移。最后，采用RAW图像格式采集并关闭自动曝光和自动白平衡功能，可有效避免因相机算法造成的非线性失真，从而提高RGB信号的可对比性和可重复性。

在数据处理方面，可通过对原始RGB数据进行归一化处理或转换至设备无关的色彩空间以降低不同成像设备及成像条件差异对结果的影响。同时进一步通过背景扣除、空白校正及噪声平滑处理有效提升信号稳定性。在低浓度区间，针对信号方差随浓度变化而产生的异方差问题，可采用加权最小二乘法进行拟合，并结合*R*²、均方根误差及残差分布等统计指标对模型性能进行综合评价。此外，为验证取色信号与实际光学响应之间的内在对应关系，应结合紫外-可见吸收光谱分析和透射电子显微镜或动态光散射表征，以揭示LSPR峰位移动与粒径、形貌演化之间的关联。通过实验结果与理论模拟的对比结合（如基于Mie理论或离散偶极近似模型），可在理论层面阐明Ag NPs在刻蚀过程中的光学响应机制，从而为实验观察结果提供更为坚实的支撑。

总体而言，尽管红色通道在该体系中展现出较高的灵敏度与良好的线性响应，但其拟合精度仍存在一定不足，表明其在定量回归建模中可能受到信号噪声或非线性干扰的限制。相较之下，蓝色通道在信号稳定性与线性拟合精度方面更具优势，更适用于高精度定量分析。而红色与绿色通道可作为辅助变量，通过多通道数据融合或多元回归校正，有助于抑制系统误差并提高整体检测的准确性与稳健性。通过严格控制光源条件与取色位置、改进样品封闭条件并优化数据处理与验证策略，可显著提高拟合线性度与检测可靠性，从而使基于手机取色法的SO_2_比色检测在保持低成本优势的同时，具备更高的科学严谨性与工程应用潜力。

### 3.3 3组平行样品检测结果

3组平行待测样品在蓝色通道的颜色强度值分别为142、143和144，利用蓝光通道对应的标准曲线将每个样品的RGB信号转换为浓度值，分别为0.50、0.46和0.41 mmol/L。对3组平行样品的浓度进行算术平均，得到平均浓度为0.46 mmol/L。

## 4 实验的组织实施及教学反思

### 4.1 实验的组织与实施

本课程围绕智能手机比色法结合HS-SDME为核心，实现了SO_2_气体污染物的定量分析实验设计，涵盖Ag NPs的合成、微量溶液配制、光学信号获取及定量分析等核心环节。课程总计8学时，包括2学时理论课程和6学时实验实践。在理论学习阶段，教师系统讲授纳米材料的基本概念、Ag NPs的理化特性及其在环境检测中的应用，同时结合溶液化学、光谱分析及数据处理等内容，引导学生理解实验原理与方法背景，通过案例分析、启发式提问与课堂讨论，强化学生的科学思维和科研伦理意识。

实验实践环节重点突出实验操作规范性、可重复性与科学性。针对SO_2_挥发吸收、Ag NPs液滴制备及颜色信号采集等关键步骤，课程提供详细操作规范，并设置标准曲线绘制环节，结合对照实验，使学生理解实验数据的可靠性及潜在误差来源。在实验操作指导中，教师强调环境样品前处理的重要性，使学生掌握从样品采集、富集到数据分析的完整链条。此外，在实验操作过程中特别强调对颜色变化的客观化分析。由于在SO_2_刻蚀Ag NPs的反应过程中，颜色变化往往较为细微，单凭肉眼难以准确区分不同浓度对应的颜色差异，因此课程专门设置了“感官观察与仪器比对”环节，使学生认识到人眼分辨率的天然局限性。通过将肉眼观察结果与智能手机比色系统获取的数字化信号进行对比分析，学生能够明确理解：即便颜色差异不明显，借助仪器提取RGB参数即可显著提升定量区分能力，从而确保检测的准确性与灵敏度。该环节有效强化了“主观感知不足以支撑环境分析，科学测量必须依赖仪器化手段”的核心理念，有助于学生建立正确的分析化学认知框架。

课程设计还充分考虑学生能力差异与学习深度，采用分层引导策略：基础操作模块侧重实验安全、准确性与规范化；高阶探究模块鼓励学生自主设计实验方案、优化操作条件，并对实验结果进行统计分析与反思讨论。实验中，学生不仅需完成Ag NPs制备、SO_2_吸附与刻蚀反应、颜色采集及数据分析，还需针对实验结果讨论方法局限性、数据波动原因及优化策略，从而培养科学分析能力、问题解决能力及科研素养。通过小组汇报与结果分享环节，学生进一步锻炼团队协作和学术表达能力。

总体而言，本实验在教学中兼顾操作严谨性和数据可靠性，同时突出创新性与跨学科应用价值，使本科生能够深入理解环境样品分析的完整流程，掌握纳米材料与智能终端分析技术的结合应用，并在实践中培养科学探究精神、创新思维、社会责任感及专业使命感。这不仅提升了实验课程的教育质量，也为学生未来的科研实践和职业发展奠定了坚实基础。

### 4.2 教学反思

在开放实验课程实施过程中，教师需充分平衡理论指导与学生自主探索的关系。本实验以智能手机比色法检测SO_2_气体污染物为主题，涵盖纳米材料合成、微量萃取、颜色信号采集及定量分析等多个环节，其跨学科特性要求学生具备化学基础知识、数据处理能力及一定的科研素养。因此，本实验的教学重点在于：一是掌握纳米材料制备与微萃取操作技能；二是理解智能手机比色法的原理及颜色信号的定量分析方法；三是能够结合实验数据进行科学推理和结果解释，从而建立系统的实验逻辑思维。教学过程中应根据学生能力进行分层引导：既要确保基础操作规范和实验安全，又要给予学生充分自主设计和创新尝试的空间，从而培养学生发现问题与解决问题的能力。

与此同时，本实验存在一定的教学难点。首先，微萃取条件、纳米材料刻蚀效率及颜色信号稳定性等关键参数对实验结果影响显著，学生需理解并掌握其调控方法。其次，实验依赖智能手机图像采集与软件数据处理，操作规范性直接影响数据准确性，因此有必要在教学中强调数据采集流程的标准化、颜色校正方法的规范化及统计分析方法的科学应用。此外，实验跨学科特性要求学生将理论知识应用于实践，如理解化学反应原理、环境样品复杂性及污染物富集机制，这也对学生的综合分析能力提出了挑战。

针对上述教学重点与难点，课程实施过程中应采用分层引导策略：在保证基础操作规范和实验安全的前提下，鼓励学生自主设计实验方案、优化操作条件并进行创新尝试。同时，通过案例分析、实验讨论与数据对比，教师可引导学生构建科学逻辑框架、培养系统思维及跨学科整合能力。课程还应结合环境科学与社会实践，引导学生关注实验方法在不同环境样品中的适用性及局限性，如气态污染物浓度波动、基体干扰和现场复杂性，从而强化科研伦理意识、社会责任感与课程思政的有机融合。通过这种教学模式，学生不仅能够掌握核心实验技能和跨学科知识，还能在实践中提升创新能力、科研素养与综合职业素质，为未来科研实践与职业发展奠定坚实基础。

## 5 结语

本开放性实验课程充分利用了校级及院级公共实验平台的相关资源，使相关专业学生能够全程参与实验项目。实验以SO_2_作为无机气体污染物的代表，成功将智能手机比色法与HS-SDME相结合，实现了对环境样品中SO_2_的快速、灵敏检测。实验结果验证了Ag NPs结合智能手机比色法在环境检测中的可行性，展示了其在大气污染分析领域的应用前景。通过智能手机识别不同分析物浓度下的光学信号，软件自动生成标准曲线并测定未知样品浓度。本课程在保证检测的高灵敏度和快速信号响应的同时，还展现了实验操作简便性的优势。课程在教学设计上具有多方面的创新与特色：一方面，通过构建微型化的样品处理与检测实验体系，并结合纳米材料的设计制备，使学生能够充分接触和掌握前沿环境分析技术；另一方面，HS-SDME方法有效降低了样品基体干扰，为无机气体污染物的现场快速分析提供了实践依据，充分体现了实验课程的科学性和应用价值。此外，利用智能手机作为便携式分析平台，将信息技术与化学实验紧密结合，不仅增强了实验的趣味性和可操作性，也体现了绿色环保和资源节约的理念。综上所述，本课程通过开放性实验的模式，将理论知识、先进技术与实际应用有机融合，不仅提升了学生的实验技能和创新能力，还培养了学生的跨学科综合应用能力和环境意识。课程实验具有操作简便、时间短、成本低且可实时分析的特点，能够有效激发学生的学习兴趣与科研探索精神，为学生在环境监测、分析化学等相关领域的科研实践和职业发展奠定了坚实基础，同时彰显了开放实验在现代高等教育实验教学中的重要价值与创新意义。
